# Sociodemographic Characteristics of Infants Receiving Nirsevimab

**DOI:** 10.1001/jamanetworkopen.2025.4341

**Published:** 2025-04-09

**Authors:** Salomé Boutin, Marion Bertrand, Jérémie F. Cohen, Mahmoud Zureik, Martin Chalumeau, Marie-Joëlle Jabagi

**Affiliations:** 1 EPI-PHARE Scientific Interest Group in Epidemiology of Health Products, French National Agency for Medicines and Health Products Safety, French National Health Insurance, Saint-Denis, France; 2 Department of General Pediatrics and Pediatric Infectious Diseases, Necker-Enfants Malades University Hospital, Assistance Publique-Hôpitaux de Paris, Université Paris Cité, Paris, France

## Abstract

This cohort study investigates inequalities by sociodemographic group in the receipt of nirsevimab immunization by infants in France from 2023 to 2024.

## Introduction

Health inequalities in early childhood (HIEC) contribute significantly to avoidable morbidity and mortality in high-income countries.^1^ Key mechanisms include limited access to health care, low health literacy, and clinician implicit bias.^1^ Understanding these factors is pivotal to preparing corrective actions, although their interconnections complicate this task. Universal free prevention programs allow assessment of noneconomic factors associated with HIEC.

Respiratory syncytial virus infection is a cause of early childhood morbidity and mortality,^2^ and until the approval of nirsevimab in 2022, no universal prevention programs existed.^3^ The first immunization campaign with nirsevimab began in France in September 2023, with a free single dose recommended for infants born after February 6, 2023.^4^ This provided an opportunity to evaluate noneconomic factors associated with HIEC. This study explored potential sociodemographic inequalities associated with nirsevimab uptake during this campaign.

## Methods

Using the French National Health Data System, this cohort study included all infants eligible for nirsevimab born February 6 to September 15, 2023 (eAppendix in Supplement 1). The event of interest was outpatient administration of a single-dose nirsevimab immunization from September 15, 2023, to January 31, 2024. However, because of national shortages during this initial campaign, only a fraction of eligible children received passive immunization.

We compared characteristics of immunized and unimmunized infants, including general characteristics (sex, gestational age, birth weight and period, and social security affiliation type), individual socioeconomic indicators (type of birth hospital, complementary solidarity health insurance status, and consultations in maternal and child welfare centers), and indicators of sociogeographical inequities (region of residence, residential municipality–related indicators of deprivation [French Deprivation Index], and health care accessibility [General Practitioners’ Localized Potential Accessibility]) (eMethods in Supplement 1). We analyzed associations of demographic and socioeconomic indicators with nirsevimab receipt using logistic regression to obtain crude and adjusted odds ratios (aORs) and 95% CIs.

By the permanent regulatory access to the National Health Data System granted to EPI-PHARE, this study did not require specific authorization from the National Commission on Informatics and Liberty, and informed consent was not required. We followed the STROBE reporting guideline. Significance was defined as a 2-tailed *P* < .05.

## Results

Of 328 131 infants in the study, 42 082 infants (12.8%) received nirsevimab (mean [SD] age at administration, 4.8 [2.3] months). Infants who were male (aOR vs female, 1.07 [95% CI, 1.05-1.10]) and born very preterm (aOR vs full term, 2.07 [95% CI, 1.82-2.37]), and in June or July (aOR vs February or March, 1.69 [95% CI, 1.64-1.74]) were more likely to be immunized.

Socioeconomic indicators were associated with nirsevimab immunization. On an individual level, infants whose parents had complementary solidarity health insurance (aOR, 0.38 [95% CI, 0.37-0.39]), were covered by the agricultural social security scheme (aOR vs the general scheme, 0.86 [95% CI, 0.79-0.93]), and received consultation in maternal and child welfare centers (aOR, 0.78 [95% CI, 0.75-0.82]) and infants born in public hospitals (aOR, 0.81 [95% CI, 0.79-0.83]), living in the most deprived municipalities (quartile 5 [Q5] vs Q1: aOR, 0.41 [95% CI, 0.39-0.42]), or with lower accessibility to general practitioners (Q1 vs Q5: aOR, 0.60 [95% CI, 0.57-0.62]) had significantly lower odds of immunization. We observed regional disparities, with higher immunization coverage in northern France (Figure).

**Figure.**
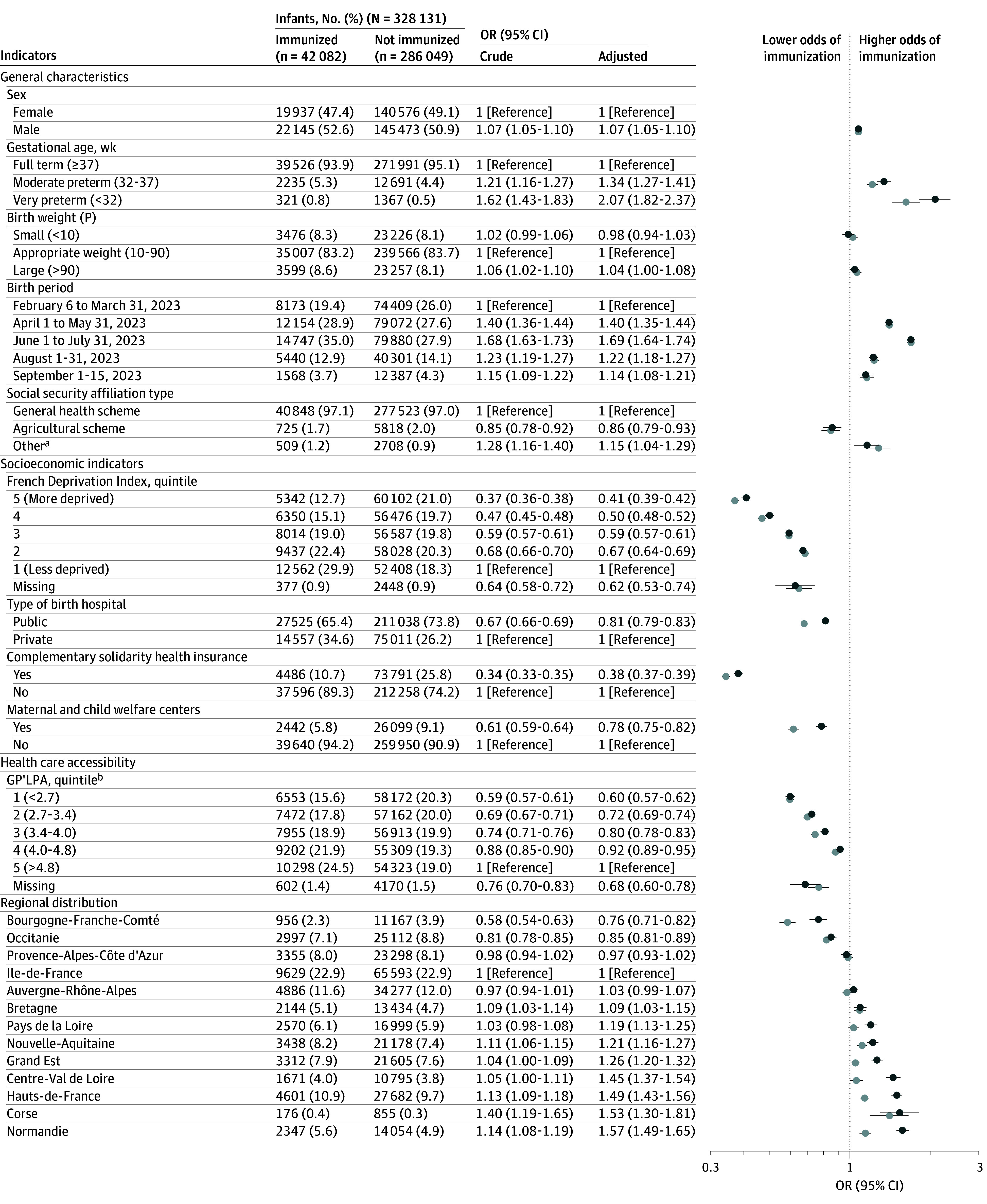
Association Between Socioeconomic and Health Care Accessibility and Outpatient Nirsevimab Immunization P indicates percentile for gestational age; OR, odds ratio. ^a^The other social security affiliation type concerns specific groups, such as seafarers; employees of the French National Railway Company, Paris Transit Authority, and Electricity and Gas; notary employees and clerks; and members of the National Assembly and Senate. ^b^GP'LPA indicates general practitioners’ localized potential accessibility in number of consultations per capita in 2022. The LPA is a continuous indicator and was categorized using quintiles calculated from the study population.

## Discussion

This cohort study found significant socioeconomic and geographical disparities in nirsevimab immunization rates during France’s first free outpatient immunization campaign. Infants born in June or July 2023 showed higher immunization rates, likely due to timely recommendations and increased parental awareness of their risk. Disparities occurred among socioeconomically deprived infants and those residing in areas with limited health care access. Geographic disparities may have been exacerbated by supply shortages, disproportionately impacting regions with higher deprivation. A study limitation is the lack of health literacy indicators. While similar inequalities have been noted in other pediatric preventive treatments globally,^5,6^ this study uniquely addresses them in a free preventive context. Transitioning to a subsidized model could worsen these disparities, underscoring the need for universal free programs to ensure equitable access to immunization. These findings emphasize the need for targeted interventions and policies to prioritize health equity for at-risk populations.

## Notes

Supplement 1.
eAppendix.

eMethods.

eReferences

Supplement 2.
Data Sharing Statement
