# Supplementary material for: Sociodemographic Characteristics of Infants Receiving Nirsevimab
Source: JAMA Netw Open. 2025 Apr 9;8(4):e254341. doi: 10.1001/jamanetworkopen.2025.4341 (PMC11983228; doi:10.1001/jamanetworkopen.2025.4341)
Supplement: Supplement 1. — eAppendix. eMethods. eReferences [file jamanetwopen-e254341-s001.pdf]

Supplemental Online Content

Boutin S, Bertrand M, Cohen JF, Zureik M, Chalumeau M, Jabagi M-J. Sociodemographic characteristics of infants receiving nirsevimab. *JAMA Netw Open*. 2025;8(4):e254341.
doi:10.1001/jamanetworkopen.2025.4341

eAppendix.

eMethods.

eReferences.

This supplemental material has been provided by the authors to give readers additional information about their work.

© 2025 Boutin S et al. *JAMA Network Open*.

eAppendix.

Abbreviations

ANSM, French National Agency for the Safety of Medicines and Health Products

ATC, Anatomical Therapeutic Chemical Classification System

EPI-PHARE, Scientific Interest Group in Epidemiology of Health Products

CNAM, French National Health Insurance

CNIL, National Commission on Informatics and Liberty

FDep, French Deprivation Index

PMSI, Medicalization of Information Systems Program

RSV, Respiratory Syncytial Virus

SNDS, National Health Data System

Context

French nirsevimab immunization program

For the 2023-2024 RSV season, passive immunization with nirsevimab has been recommended for all children born after February 6, 2023. The campaign started on September 15, 2023. The recommendation was to provide nirsevimab to newborns before leaving the maternity ward. For eligible infants who had already been discharged from the maternity ward by September 15, 2023, nirsevimab was provided free of charge in the outpatient setting by community pharmacies.

Respiratory Syncytial Virus (RSV) season in France

The 2023–2024 RSV season started in October 2023 and ended around the end of January, with a peak between November 15 and December 15, 2023.

© 2025 Boutin S et al. *JAMA Network Open*.

eMethods

Data source

The National Health Data System (SNDS) is a collection of pseudonymized databases providing information on the entire French population. It is primarily based on the National Information System of Inter-regime Health Insurance (SNIIRAM), which includes both data on mandatory health insurance reimbursements and data from healthcare establishments collected through the Program for the Medicalization of Information Systems (PMSI). The SNDS is further enriched by databases on medical causes of death, disability and COVID-19 vaccinations and tests.

This study was conducted in accordance with Articles L.1461-3 and R. 1461-11 and following the French Public Health Code, amended by Decree No. 2021-848 of June 29, 2021, related to the processing of personal data known as the "National Health Data System" (SNDS). EPI-PHARE accesses SNDS data through the permanent regulatory access of its supervisory agencies, the ANSM and the CNAM, in accordance with the provisions of Decree No. 2016-1871 of December 26, 2016, related to the processing of personal data known as the "National Health Data System," Articles R.1461-13 and R.1461-14 of the Public Health Code, and CNIL deliberation No. 2016-316 of the National Commission on Informatics and Liberty (CNIL). In accordance with the permanent regulatory access granted to EPI-PHARE via the ANSM and the CNAM, this work did not require specific authorization from the CNIL. This study was previously declared on the EPI-PHARE register of studies requiring the use of SNDS under reference T-2024-07-525. All queries were performed by individuals duly authorized with profile 30 or 108.

Study population

All children born between February 6, 2023, and September 15, 2023, and having completed their birth stay in maternity before September 15, 2023, were selected; infants born after September 15, 2023 could have been immunized in the maternity ward and thus were excluded, since in-hospital administration information was unavailable in our database. Among these infants, we included those for which the birth stay could be found in the Medicalization of Information Systems Program (PMSI). Then, we excluded children born in the overseas departments and regions because the start dates of the immunization campaign and the dates of the RSV epidemic did not align with those of the French mainland. Then, we

excluded infants with missing data for at-birth variables (gestational age and weight at birth) and we excluded the twins for technical reasons specific to the SNDS. Infants who already had an RSV immunity (who received Palivizumab or already had bronchiolitis) were excluded as well. Finally, we also excluded children who died before leaving the birth stay or who died before September 15, 2023.

Outcome

The outcome was an immunization with one injection of nirsevimab, which was assessed using the ATC code J06BD08.

Covariates

General characteristics included sex, gestational age, birth weight, birth period, and social security affiliation type (General health scheme / Agriculture scheme). The agriculture scheme is a specialized branch of France's social security system that covers individuals working in the agricultural sector. This scheme provides health, retirement, and disability benefits and reflects the distinct employment conditions in agriculture compared to the general workforce.

Individual socioeconomic indicators included type of birth hospital (Public / private), complementary solidarity health insurance status, and consultations in maternal and child welfare centers. Public Hospitals are state-operated healthcare facilities in France that provide universal access to medical services. They are a key part of the healthcare system and often serve socioeconomically disadvantaged populations due to their accessibility and affordability. Complementary Solidarity Health Insurance is a government-supported health insurance program in France designed for low-income individuals and families. It provides access to healthcare services free of charge or at a significantly reduced cost, including consultations, medications, and hospitalizations. Eligibility is determined based on income thresholds. Maternal and Child Welfare Centers are public health institutions in France, known as protection maternelle et infantile (PMI), offering free or low-cost services to support maternal and child health. These services include prenatal and postnatal care, vaccinations, and developmental monitoring, often focusing on vulnerable families.

In addition to individual socioeconomic and sociodemographic explanatory variables, two collective ones were added based on the indicated residential municipality of each infant in the database. The French

© 2025 Boutin S et al. *JAMA Network Open*.

Deprivation Index (FDep) [1] is a marker of socio-economic status based on the residence area's median household income, percentage of high school graduates in the population aged ≥15 years, percentage of manual workers in the labor force, and unemployment in the individual's city of residence. The General Practitioners' Localized Potential Accessibility (GP'LPA) [2] is a density of general practitioner' consultations available per year per individual in each municipality standardized by age. It was created with data from several national institutions. Since the GP'LPA is a continuous indicator, we discretized it into quintiles, based on calculations from our study population.

Statistical analysis

All analyses were performed using the R software, version 4.3.2.

© 2025 Boutin S et al. *JAMA Network Open*.

eReferences

1. Rey G, Jougla E, Fouillet A, Hémon D. Ecological association between a deprivation index and mortality in France over the period 1997 - 2001: variations with spatial scale, degree of urbanicity, age, gender and cause of death. BMC Public Health. 2009;9:33.

2. Lucas-Gabrielli V, Mangeney C. Comment améliorer les mesures d'accessibilité aux soins pour mieux cibler les zones sous-denses ? [How can accessibility measures be improved to better target underserved areas?]. Rev Epidemiol Sante Publique. 2019 Feb;67 Suppl 1:S25-S32. French. doi: 10.1016/j.respe.2018.12.061. Epub 2019 Jan 11.

© 2025 Boutin S et al. *JAMA Network Open*.
