# Supplementary material for: Sociodemographic Characteristics of Infants Receiving Nirsevimab
Source: JAMA Netw Open. 2025 Apr 9;8(4):e254341. doi: 10.1001/jamanetworkopen.2025.4341 (PMC11983228; doi:10.1001/jamanetworkopen.2025.4341)
Supplement: Supplement 2. — Data Sharing Statement [file jamanetwopen-e254341-s002.pdf]

Data Sharing Statement

Boutin. Sociodemographic Characteristics of Infants Receiving Nirsevimab. *JAMA Netw Open*. Published online April 9, 2025. doi:10.1001/jamanetworkopen.2025.4341

Data
Data available: No

Additional Information
Explanation for why data not available: According to data protection and the French regulation, the authors cannot publicly release the data from the French national health data system (SNDS). However, any person or organization, public or private, for-profit or non-profit, is able to access SNDS data upon authorization from the French Data Protection Office (CNIL) to carry out a study, a research, or an evaluation of public interest.
